# Transcriptional effects of gene dose reduction

**DOI:** 10.1186/2042-6410-5-5

**Published:** 2014-03-03

**Authors:** Zhen-Xia Chen, Kseniya Golovnina, Hina Sultana, Satish Kumar, Brian Oliver

**Affiliations:** 1 Laboratory of Cellular and Developmental Biology, National Institute of Diabetes and Digestive and Kidney Diseases, National Institutes of Health, Bethesda, MD 20892-8028, USA

**Keywords:** Sex, Monosomy, X chromosome, Dosage compensation, Buffering, Feedback, Feed-forward, MSL complex

## Abstract

Large-scale gene dose reductions usually lead to abnormal phenotypes or death. However, male mammals, *Drosophila*, and *Caenorhabditis elegans* have only one X chromosome and thus can be considered as monosomic for a major chromosome. Despite the deleterious effects brought about by such gene dose reduction in the case of an autosome, X chromosome monosomy in males is natural and innocuous. This is because of the nearly full transcriptional compensation for X chromosome genes in males, as opposed to no or partial transcriptional compensation for autosomal one-dose genes arising due to deletions. Buffering, the passive absorption of disturbance due to enzyme kinetics, and feedback responses triggered by expression change contribute to partial compensation. Feed-forward mechanisms, which are active responses to genes being located on the X, rather than actual gene dose are important contributors to full X chromosome compensation. In the last decade, high-throughput techniques have provided us with the tools to effectively and quantitatively measure the small-fold transcriptional effects of dose reduction. This is leading to a better understanding of compensatory mechanisms.

## Review

Gene dose reductions are universal and have been studied in a variety of eukaryotes, including mammals [1-6], birds [7], flies [8,9], worms [10], and yeasts [11]. It is thought that gene dose reductions are tolerated or deleterious based on how out of balance the genome is following a dose change [12]. Although translation is the closest direct measurement of levels of protein function [13,14], which is what should count in the genic balance hypothesis, transcription (mRNA abundance) is typically measured. The importance of gene dose can be illustrated by co-expression levels of genes encoding proteins that are part of the same multi-subunit complex [15]. This balance requirement may extend beyond genes encoding complex members. Although dose reductions of only a few single genes are directly deleterious (haploinsufficient), the subtle effects of dose reduction for most genes are cumulative and may eventually lead to the collapse of the regulatory network. In flies, deletions reduce viability when they reduce the gene dose for >1% of the genome and cause death when they reduce the gene dose for >3% of the genome [16]. This is not the result of rare haploinsufficient loci scattered in the genome, as large deletions are lethal even when smaller deletions subdivided from them are viable. These data indicate that the deleterious effects of most large deletions result from the collapse of regulatory networks.

Natural monosomy of the X chromosome is a remarkable exception to the rule of detrimental effects of large gene dose imbalance. The viability of X chromosome monosomies (i.e., males) is due to compensatory mechanisms evolved to solve the genetic imbalances brought by the emergence of sex chromosomes. Female mammals and *Drosophila* have two X chromosomes, and males have one X chromosome and one Y chromosome. Hermaphrodite *Caenorhabditis elegans* have two X chromosomes, and males have one X chromosome. While still somewhat controversial, it appears that all three systems cope with monosomy by a twofold upregulation of highly expressed X-linked genes in males to balance gene expression between autosomes and sex chromosomes [17,18]. However, they have quite different strategies to balance female X chromosome expression in the face of male dosage compensation [19]. In *Drosophila*, upregulation of X chromosome expression is limited to males. In mammals, one X chromosome is globally inactivated in females, while in *C. elegans*, both X chromosomes in hermaphrodites are repressed by half [18]. The common feature is that X chromosome expression relative to autosomes is maintained in both sexes. X chromosome dosage compensation is often stated as a way to balance gene expression between the sexes, but it is genic balance within a sex that must be rigorously regulated.

### Dosage compensation on autosomes and X chromosomes

One of the problems with thinking about X chromosome dosage compensation in males is our rudimentary understanding of the relationship between gene dose and expression when a non-sex chromosome suffers a deletion. Effective measurements of minor-fold difference in expression are needed to track the response to gene dose reduction on autosomes. We need to understand this non-specialized response to dose in order to determine if X chromosome dosage compensation is layered over a general response or if X chromosome dosage compensation replaces a generic dosage compensation system.

Cell lines and cancer cells are good models for studying the relationship between gene dose and expression, because they are selected for growth and accumulate gene dose changes that would otherwise be selected against in the organism [9,20]. In the absence of compensation, genes with a 50% dose reduction would have 50% of the expression. However, in *Drosophila*, partial transcriptional compensation for dose reductions occurs in some, but not all aneuploid cell lines [9,21]. Generally, as gene dose increases, the expression per dose decreases and *vice versa*. Interestingly, gene dose reduction seems to be marginally better compensated than gene dose increase. For example, if there were no compensation, the expression level of one-dose genes and three-dose genes would be 50% and 150%, respectively, of the diploid baseline; if there were full compensation, their expression levels would both be 100%. However, in the diploid *D8* cell line, the expression level of one-dose genes and three-dose genes are 75% and 133%, respectively [9]. Interestingly, there are cell line-specific differences in levels of compensation in *Drosophila* cell lines*.* The cause of this variability is unknown, but it does suggest that compensation is not a universal effect. Perhaps, the levels of compensation are determined by the selection against deleterious effects of imbalance, where higher compensation would have evolved for more deleterious dose changes in these cells. Similarly, the higher compensation for one-dose genes than that for three-dose genes suggests that dose reduction is more deleterious to cells than dose increase.

The genetic model organisms allow for careful monitoring of baseline dosage compensation responses. For example, the DrosDel Deletion Collection has a set of *Drosophila melanogaster* lines with mapped deletions tiling the genome in a common genetic background [22]. Partial transcriptional compensation for one-dose genes is also observed in these DrosDel lines. Microarray analysis has found that the expression level of one-dose genes is approximately 64% of the two-dose gene expression [23,24]. While there may be compression of expression ratios in array studies resulting in over-estimation of compensation, and while the measurement of lowly expressed genes are problematic in both array and RNA-Seq experiments, the common trend is greater expression than expected if the relationship between DNA dose and RNA was 1:1. The consistent partial compensation observed in different studies focused on different deletions may be the result of a homogeneous dose response for all one-dose genes in whole animals. This model is essentially a dose damage response, where an unknown complex mediating a fixed twofold increase in gene expression recognizes a deletion and applies a correction to gene expression. If this hypothesis is correct, we would expect a normal distribution of expression change of one-dose genes, as observed in one analysis [23]. While no mechanism for a dose damage response is known, it may be analogous to painting of fourth (POF). POF is a compensation protein that decorates the very small (approximately 70 genes) chromosome 4 in *Drosophila*, which is often lost resulting in monosomy [25]. The expression of non-ubiquitously expressed genes on the fourth chromosome has been shown to be compensated by POF in flies, and the lack of POF causes lethality, exactly as expected for a dose damage response [23]. A competing model is that observed levels of compensation are the result of the characteristics of gene-specific dose responses. This is a gene regulation or transcriptional homeostasis model. If this hypothesis is correct, we would expect the dose response is not uniform across genes, as observed in the extended tails towards better compensation in the distribution of expression change of one-dose genes in one analysis [24]. Moreover, if the dose response is heterogeneous, the genetic pathways including the gene with altered gene dose should influence compensation. The consequences of incomplete or absent dosage compensation are reduced expression of the one-dose genes [17,21,24,26,27] and indirect propagating effects on two-dose genes through regulatory networks [24,27-30]. Twenty-one different lines of *Drosophila*, each with unique small deletions collectively covering 5% of genes, resulted in the expression change in 80% of genes in the entire genome [24]. In other words, the effect of a dose change is not restricted to the genes with reduced copy. There is extensive propagation of dose effects into the entire gene expression network. The fact that compensation for dose change in some aneuploid cells is stronger than in adult *Drosophila* with deletions and that other aneuploid cell lines show very poor compensation also argues against a strictly fixed dose response. Clearly, there is much work that remains to be done on exploring the response to gene dose, but these early studies suggest a way towards developing better compensation models. We can exploit current gene expression profiling models, but we need to examine more genes to see if there are gene-specific or region-specific compensation rules, determine the nature of variable compensation responses, and map out the propagation pathways to determine if they represent coherent biological pathways.

Although there is partial compensation for gene dose reduction on the autosomes, full compensation for X chromosome dose is observed in males. To solve the genetic imbalances brought by the dose reduction of X-linked genes, dosage compensation machinery needs to recognize the X chromosome (targeting), increase expression of X-linked genes in males (upregulation), and avoid the over-expression of the X chromosome genes in females (restriction). In *Drosophila*, the MSL complex is a male-specific ribonucleoprotein consisting of at least five proteins male-specific lethal 1–3 (MSL1, MSL2, and MSL3), maleless (MLE), and males absent on the first (MOF) and two non-coding RNAs (*RNA on the X* (*roX*) *1* and *2*). MSL1, MSL2, *roX1*, and *roX2* are required for targeting the X chromosome [31]. The *roX1* and *roX2* loci encode non-coding RNAs that are required for the full assembly of MSL complex, as without *roX* RNAs, MSL complex mis-localizes to centromeric heterochromatin [32]. The *roX* genes are X-linked, but when they are translocated to autosomes, high-level expression of *roX* genes still favors recruitment of MSL complex to the X chromosome [32]. However, low-level expression of autosomal *roX* genes favors local bidirectional spreading of MSL complex, suggesting that *roX* RNAs are incorporated into the MSL complex when they are transcribed [33]. It is possible that X linkage is favorable for *roX* function. MOF, a histone acetyltransferase that modifies histone H4 at lysine 16 (H4K16), performs the upregulation function. H4K16 acetylation not only activates transcription [34] but also loosens chromatin structure globally [35] and thus is considered to de-repress X chromosome genes in males. An alternative, less well-accepted model suggests that MOF is a global activator of transcription [36] and that the MSL complex sequesters MOF to the X chromosome to prevent the upregulation of autosomal genes [37]. MOF does bind to autosomal sites in both sexes [38], raising the possibility that male-specific MSL complexes and non-sex-specific MOF complexes exist and compete. Both the recruitment of activator and sequestration models balance X and autosome expression in both sexes. Sex-specific regulation of MSL2 is also required to restrict MSL action to males. The translation of *msl2* mRNA is inhibited by Sex-lethal (SXL), which responds to X chromosome number resulting in SXL expression and dosage compensation suppression only in females [39]. Since MSL complex does not locate to the X chromosome without MSL2, this ensures that upregulation of X chromosome genes is limited to males.

**Figure 1:**
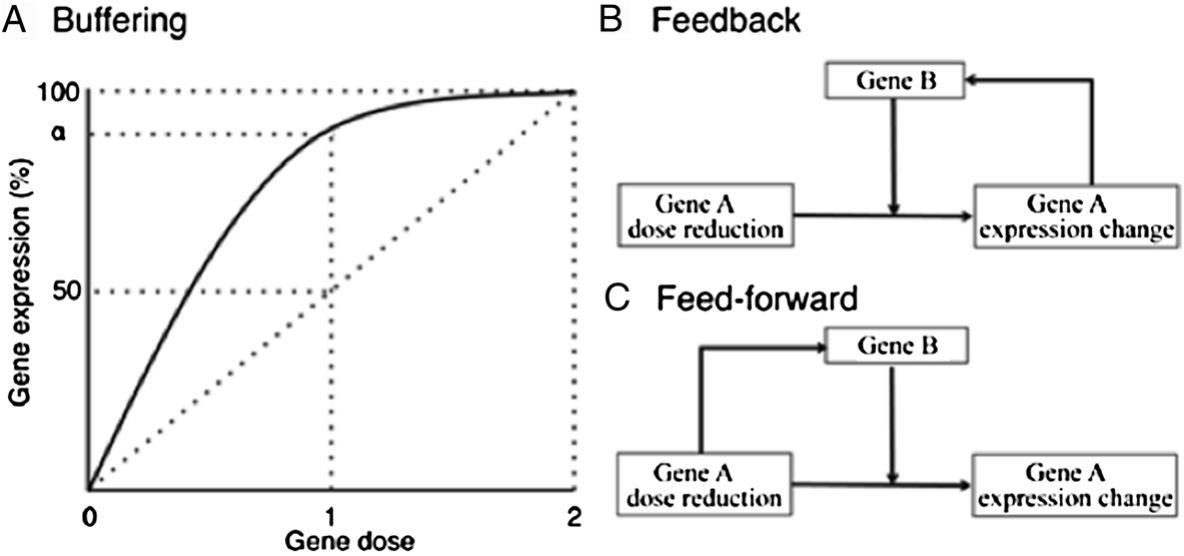
**Mechanisms resulting in dosage compensation. (A)** Buffering results in non-linear relationship between gene dose and expression due to system properties such as kinetics. This does not result in full compensation but can dampen the effect of gene dose. **(B)** Feedback requires the generation and detection of an error in expression followed by intervention to correct the error. **(C)** Feed-forward, as it relates to X chromosome dosage compensation, results in the upregulation of all X chromosome genes regardless of the expression level.

Autosomal and X chromosome dosage compensation may be mediated by one or more mechanisms, including buffering, feedback, and feed-forward that can be used to ameliorate the effect of gene dose reduction in living systems [21]. Buffering is the passive absorption of effects caused by dose reduction. For example, gene transcription may be considered as a chemical reaction obeying mass action kinetics, where the template gene is a catalyst and mRNAs are products. The flux (expression level) through the reaction is dependent on kinetic parameters and should not follow a linear relationship (Figure 1A) [40]. Therefore, the 50% dose reduction from two doses to one dose should reduce expression <50% and thus result in partial compensation. However, the effect of buffering can only lower the expression change caused by dose reduction, but not eliminate it. At least some genes are overexpressed as a result of decreased gene dose, which cannot be explained by buffering [24]. Feedback is an action triggered by the expression change to restore the balance. For example, the dose reduction of gene A leads to its expression reduction, which recruits regulator B to activate gene A and thus demonstrate compensation (Figure 1B). Feedback, as error-controlled regulation, takes action when the expression changes and thus can respond to expression change caused by any disturbance. Moreover, the effect of feedback is gene specific and may result in over-, full, partial, no, or anti-compensation. Considering the heterogeneous dose response for most dose reductions [24], feedback is likely to play an important role in partial compensation. However, feedback might not be sufficient to cope with the large number of dose changes due to monosomy of the X chromosome. Feed-forward is another action to restore the gene expression with reduced dose. For example, the regulator B may recognize and target the one-dose gene A and suppress A's expression change before the fact (Figure 1C). Feed-forward anticipates the dose reduction before it influences expression change and thus may be crucial for full compensation; unlike in the case of feedback, the correction occurs before the expression error is generated. Outside of the well-studied MSL-mediated feed-forward system, we have a very poor understanding of the contributions these mechanisms make to X chromosome compensation. Indeed, there may even be undiscovered feed-forward mechanisms to explain the MSL-independent full X chromosome compensation in the *Drosophila* male germline for example [17,18]. We do not understand how such feed-forward themes evolved, but MSL components such as MOF are widely deployed in other species as general activators of transcription, suggesting that such general regulators can be co-opted for chromosome-wide duties.

### Why is X chromosome dosage compensation better than autosomal compensation?

Full compensation, as observed for the *Drosophila* X chromosome, is an elegant solution evolved to eliminate the deleterious effects of dose reduction and genic imbalance, and is clearly important for male viability. If dosage compensation is so valuable, then full compensation for deletions should also be favored. Why is compensation for deletions so poor? In at least some situations, gene dose is regulated in order to produce large amounts of product. For example, there is differential amplification of genes in terminally differentiated *Drosophila* cell, such as those encoding eggshell proteins [41]. Full dosage compensation would undo these advantageous dose differences. This is not restricted to *Drosophila.* A high rate of aneuploidy, including dose reduction, appears to be part of normal hepatocyte function in the mammalian liver [42]. Imprinting can be viewed as an epigenetic dose reduction and is widespread in many species [43]. Finally, there are a few X chromosome genes that escape dosage compensation [43]. Given the multiple cases of regulated dose differences, it seems likely that universal and full compensation would be selected against.

One of the main differences between a sex chromosome and autosomal monosomy is the long path to compensation in the X chromosome lineage. Deletions and autosomal monosomies are sporadic and generally have a very short duration in the gene pool of any given species, while sex chromosome evolution is a driven process that persists in related species over many millions of years. It is possible that only sex chromosomes are fully compensated because it takes a great deal of time to fully fine-tune the process. For example, in *Drosophila miranda*, there are three X chromosome fragments of differing age as a result of interchromosome fusions: XL (>60 million years old), XR (approximately 15 million years old), and neo-X (approximately 1 million years old). Only partial compensation is observed on the neo-X segment even though the MSL machinery that already evolved to control X chromosome compensation is co-opted to regulate the neo-X segment [44,45]. It seems likely that new cis-acting elements are acquired step by step over time, rather than all at once [46].

Sexual selection might also be important for the evolution of X chromosome dosage compensation. To this point, we have used sex chromosome dosage compensation as an example of full compensation; however, this is not always the case. These exceptions might be quite informative. In birds, which have ZW sex determination (ZW females and ZZ males), only partial Z chromosome dosage compensation is detected [47]. This partial compensation might be similar to the autosomal compensation observed in *Drosophila.* The ZW sex determination system dates to the dinosaurs, so we cannot explain the lack of robust compensation by the absence of fine-tuning time. We also cannot explain poor Z chromosome compensation based on the fraction of the genome linked to sex chromosomes. In *Gallus gallus*, the Z chromosome comprises 8% of the genome [16,48-52], while reduced dose for 3% of the fly genome cause death [16]. If the number of genes on the Z cannot explain poor compensation, perhaps Z had a longer time to adapt due to the peculiar nature of sex linkage, sexual selection, and mutation rate [53].

Sexually antagonistic genes, which are advantageous to one sex but deleterious to the other sex, will accumulate to improve the function of one sex with the cost of reducing function in the other sex. In XY systems, this results in some predictions. Sexually antagonistic genes that are advantageous to male would favor the linkage with male determinant on the proto-Y so that they can exert their advantageous functions in males (with proto-Y) while avoiding their deleterious functions in females (without proto-Y). With the accumulation of sexually antagonistic genes, the non-recombining region between proto-X and proto-Y expanded, and eventually, the recombination between sex chromosomes is suppressed in males but not in females [54]. The absence of recombination between proto-X and proto-Y led to the gradual degeneration of the Y chromosome by accumulation of deleterious mutations in an irreversible manner (called Muller's ratchet) or through hitchhiking by favorable mutations (called selective sweeps) [55]. First, the loss of genes on proto-Y chromosome may act as common dose reduction and be compensated through buffering and feedback in a gene-by-gene manner as the partial compensation. Later, with the degeneration of proto-Y chromosome, the number of one-dose X-linked genes increased in males [25]. In this situation, the deleterious effect of dose reduction without full compensation is large and unavoidable, and thus an active response prior to the expression change rather than a reaction to expression change is required. In this scenario, feed-forward is strongly selected for in order to restore the expression balance between autosomes and X chromosome as well as between sexes. Instead of responding to the expression change caused by dose reduction, this system responds *a priori* in the sex where the X chromosome dose is expected to be one [21]. This chromosome-wide upregulation may be achieved by a combination of partial compensation and feed-forward mechanisms [21,36], but how these mechanisms might interact is unclear.

While in both XY and ZW systems the sex chromosomes arose from an ancestral autosome pair and genes became lost on the Y and W chromosomes due to the absence of recombination with a homolog, the two different systems might have required different dosage compensation solutions. For example, in the bird population, no W chromosomes are in males, while in *Drosophila* populations, no Y chromosomes are in females. Thus, unlike the autosomes, the sex chromosomes are not under selection equally in the sexes. Both XY and ZZ males should accumulate mutations faster due to the greater number of cell divisions leading to spermatogenesis relative to oogenesis. Considering the strong male-biased mutation rate, if W-linked genes degenerate slower than Y-linked genes, then the X chromosome would face a greater dose imbalance due to faster Y chromosome gene loss, thus leading to the evolution of full compensation in XY systems and a more adaptive gene regulation response in ZW systems, such as birds [47]. In this model, the partial dosage compensation observed in birds would share many of the gene-by-gene features of autosomal dosage compensation in *Drosophila*. It would be interesting to determine the differences between Z chromosome and autosome dosage compensation in these species.

## Conclusions

There is full compensation for the one-dose X-linked genes in males, while there is generally only partial compensation for one-dose genes on the autosomes of either sex. Buffering and feedback mechanisms may underlie the partial compensation, while a feed-forward mechanism is involved in the full compensation of X-linked genes in wild-type males. This hypothesis is supported by evidence from *Drosophila*, the most well-studied model for dosage compensation. However, there are many unsolved questions: What is the exact contribution of each mechanism to full compensation? What is the MSL-independent feed-forward mechanism? How does the MSL complex achieve twofold upregulation? etc. It is crucial to untangle compensatory mechanisms and separate potential impacting factors. For example, most systematic studies for dose response focus on autosomal genes. It is still not clear whether genes on different genomic location would have different dose response. A systematic comparison between the responses of one-dose X-linked genes and one-dose autosomal genes in homogametic sex will provide us with deeper understanding of compensatory mechanisms.

## Competing interests

The authors declare that they have no competing interests.

## Authors’ contributions

BO and Z-XC conceived and wrote the manuscript. KG, HS, and SK provided intellectual input. All authors read and approved the final manuscript.

## Authors’ information

BO is the chief and ZC, KG, SK, and HS are Visiting Fellow Award recipients in the section of Developmental Genomics, Laboratory of Cellular and Developmental Biology, National Institute of Diabetes and Digestive and Kidney Diseases, National Institutes of Health, Bethesda, MD, USA.

## References

[B1] Cooper GM Nickerson DA Eichler EE Mutational and selective effects on copy-number variants in the human genome Nat Genet 2007 5 S22 S29 10.1038/ng2054 17597777

[B2] Schrider DR Hahn MW Gene copy-number polymorphism in nature Proc Biol Sci 2010 5 3213 3221 10.1098/rspb.2010.1180 20591863 PMC2981937

[B3] Perry GH Yang F Marques-Bonet T Murphy C Fitzgerald T Lee AS Hyland C Stone AC Hurles ME Tyler-Smith C Eichler EE Carter NP Lee C Redon R Copy number variation and evolution in humans and chimpanzees Genome Res 2008 5 1698 1710 10.1101/gr.082016.108 18775914 PMC2577862

[B4] Lee AS Gutierrez-Arcelus M Perry GH Vallender EJ Johnson WE Miller GM Korbel JO Lee C Analysis of copy number variation in the rhesus macaque genome identifies candidate loci for evolutionary and human disease studies Hum Mol Genet 2008 5 1127 1136 10.1093/hmg/ddn002 18180252

[B5] Liu GE Hou Y Zhu B Cardone MF Jiang L Cellamare A Mitra A Alexander LJ Coutinho LL Dell'Aquila ME Gasbarre LC Lacalandra G Li RW Matukumalli LK Nonneman D Regitano LCA Smith TPL Song J Sonstegard TS Van Tassell CP Ventura M Eichler EE McDaneld TG Keele JW Analysis of copy number variations among diverse cattle breeds Genome Res 2010 5 693 703 10.1101/gr.105403.110 20212021 PMC2860171

[B6] Chen WK Swartz JD Rush LJ Alvarez CE Mapping DNA structural variation in dogs Genome Res 2009 5 500 509 19015322 10.1101/gr.083741.108 PMC2661804

[B7] Griffin DK Robertson LB Tempest HG Vignal A Fillon V Crooijmans RP Groenen MA Deryusheva S Gaginskaya E Carre W Waddington D Talbot R Völker M Masabanda JS Burt DW Whole genome comparative studies between chicken and turkey and their implications for avian genome evolution BMC Genomics 2008 5 168 10.1186/1471-2164-9-168 18410676 PMC2375447

[B8] Emerson JJ Cardoso-Moreira M Borevitz JO Long M Natural selection shapes genome-wide patterns of copy-number polymorphism in *Drosophila melanogaster* Science 2008 5 1629 1631 10.1126/science.1158078 18535209

[B9] Lee H McManus CJ Cho D-Y Eaton M Somma P Cherbas L Resch A Powell S Zhang D May G Zhan L Andrews J Celniker S Cherbas P Przytycka T Gatti M Oliver B Graveley B MacAlpine D DNA copy number evolution in modENCODE cell lines Genome Biol 2014 in press 10.1186/gb-2014-15-8-r70 PMC4289277 25262759

[B10] Maydan JS Lorch A Edgley ML Flibotte S Moerman DG Copy number variation in the genomes of twelve natural isolates of *Caenorhabditis elegans* BMC Genomics 2010 5 62 10.1186/1471-2164-11-62 20100350 PMC2822765

[B11] Carreto L Eiriz MF Gomes AC Pereira PM Schuller D Santos MA Comparative genomics of wild type yeast strains unveils important genome diversity BMC Genomics 2008 5 524 10.1186/1471-2164-9-524 18983662 PMC2588607

[B12] Birchler JA Veitia RA Gene balance hypothesis: connecting issues of dosage sensitivity across biological disciplines Proc Natl Acad Sci U S A 2012 5 37 14746 14753 10.1073/pnas.1207726109 22908297 PMC3443177

[B13] Schwanhausser B Busse D Li N Dittmar G Schuchhardt J Wolf J Chen W Selbach M Global quantification of mammalian gene expression control Nature 2011 5 337 342 10.1038/nature10098 21593866

[B14] Lu P Vogel C Wang R Yao X Marcotte EM Absolute protein expression profiling estimates the relative contributions of transcriptional and translational regulation Nat Biotechnol 2007 5 117 124 10.1038/nbt1270 17187058

[B15] Jansen R Greenbaum D Gerstein M Relating whole-genome expression data with protein-protein interactions Genome Res 2002 5 1 37 46 10.1101/gr.205602 11779829 PMC155252

[B16] Lindsley DL Sandler L Baker BS Carpenter AT Denell RE Hall JC Jacobs PA Miklos GL Davis BK Gethmann RC Hardy RW Steven AH Miller M Nozawa H Parry DM Gould-Somero M Gould-Somero M Segmental aneuploidy and the genetic gross structure of the *Drosophila* genome Genetics 1972 5 1 157 184 4624779 10.1093/genetics/71.1.157 PMC1212769

[B17] Gupta V Parisi M Sturgill D Nuttall R Doctolero M Dudko OK Malley JD Eastman PS Oliver B Global analysis of X-chromosome dosage compensation J Biol 2006 5 1 3 10.1186/jbiol30 16507155 PMC1414069

[B18] Deng X Hiatt JB Nguyen DK Ercan S Sturgill D Hillier LW Schlesinger F Davis CA Reinke VJ Gingeras TR Shendure J Waterston RH Oliver B Lieb JD Disteche CM Evidence for compensatory upregulation of expressed X-linked genes in mammals, *Caenorhabditis elegans* and *Drosophila melanogaster* Nat Genet 2011 5 12 1179 1185 10.1038/ng.948 22019781 PMC3576853

[B19] Meller VH Kuroda MI Sex and the single chromosome Adv Genet 2002 5 1 24 11931222 10.1016/s0065-2660(02)46002-6

[B20] Davoli T Xu AW Mengwasser KE Sack LM Yoon JC Park PJ Elledge SJ Cumulative haploinsufficiency and triplosensitivity drive aneuploidy patterns and shape the cancer genome Cell 2013 5 4 948 962 10.1016/j.cell.2013.10.011 24183448 PMC3891052

[B21] Zhang Y Malone JH Powell SK Periwal V Spana E Macalpine DM Oliver B Expression in aneuploid *Drosophila* S2 cells PLoS Biol 2010 5 2 e1000320 10.1371/journal.pbio.1000320 20186269 PMC2826376

[B22] Ryder E Ashburner M Bautista-Llacer R Drummond J Webster J Johnson G Morley T Chan YS Blows F Coulson D Reuter G Baisch H Apelt C Kauk A Rudolph T Kube M Klimm M Nickel C Szidonya J Maróy P Pal M Rasmuson-Lestander A Ekström K Stocker H Hugentobler C Hafen E Gubb D Pflugfelder G Dorner C Mechler B The DrosDel deletion collection: a *Drosophila* genomewide chromosomal deficiency resource Genetics 2007 5 1 615 629 10.1534/genetics.107.076216 17720900 PMC2013729

[B23] Stenberg P Lundberg LE Johansson AM Ryden P Svensson MJ Larsson J Buffering of segmental and chromosomal aneuploidies in *Drosophila melanogaster* PLoS Genet 2009 5 5 e1000465 10.1371/journal.pgen.1000465 19412336 PMC2668767

[B24] Malone JH Cho DY Mattiuzzo NR Artieri CG Jiang L Dale RK Smith HE McDaniel J Munro S Salit M Andrews J Przytycka TM Oliver B Mediation of *Drosophila* autosomal dosage effects and compensation by network interactions Genome Biol 2012 5 4 r28 10.1186/gb-2012-13-4-r28 22531030 PMC3446302

[B25] Stenberg P Larsson J Buffering and the evolution of chromosome-wide gene regulation Chromosoma 2011 5 3 213 225 10.1007/s00412-011-0319-8 21505791 PMC3098985

[B26] Lundberg LE Figueiredo ML Stenberg P Larsson J Buffering and proteolysis are induced by segmental monosomy in *Drosophila melanogaster* Nucleic Acids Res 2012 5 5926 5937 10.1093/nar/gks245 22434883 PMC3401434

[B27] Henrichsen CN Chaignat E Reymond A Copy number variants, diseases and gene expression Hum Mol Genet 2009 5 R1 R8 10.1093/hmg/ddp011 19297395

[B28] Dermitzakis ET Stranger BE Genetic variation in human gene expression Mamm Genome 2006 5 6 503 508 10.1007/s00335-006-0005-y 16783632

[B29] Reymond A Henrichsen CN Harewood L Merla G Side effects of genome structural changes Curr Opin Genet Dev 2007 5 381 386 10.1016/j.gde.2007.08.009 17913489

[B30] Henikoff S Dosage-dependent modification of position-effect variegation in *Drosophila* Bioessays 1996 5 5 401 409 10.1002/bies.950180510 8639163

[B31] Gelbart ME Kuroda MI *Drosophila* dosage compensation: a complex voyage to the X chromosome Development 2009 5 9 1399 1410 10.1242/dev.029645 19363150 PMC2674252

[B32] Meller VH Rattner BP The roX genes encode redundant male-specific lethal transcripts required for targeting of the MSL complex EMBO J 2002 5 5 1084 1091 10.1093/emboj/21.5.1084 11867536 PMC125901

[B33] Kelley RL Lee OK Shim YK Transcription rate of noncoding roX1 RNA controls local spreading of the *Drosophila* MSL chromatin remodeling complex Mech Dev 2008 5 1009 1019 10.1016/j.mod.2008.08.003 18793722 PMC2659721

[B34] Akhtar A Becker PB Activation of transcription through histone H4 acetylation by MOF, an acetyltransferase essential for dosage compensation in *Drosophila* Mol Cell 2000 5 367 375 10.1016/S1097-2765(00)80431-1 10882077

[B35] Robinson PJ An W Routh A Martino F Chapman L Roeder RG Rhodes D 30 nm chromatin fibre decompaction requires both H4-K16 acetylation and linker histone eviction J Mol Biol 2008 5 816 825 10.1016/j.jmb.2008.04.050 18653199 PMC3870898

[B36] Prestel M Feller C Becker PB Dosage compensation and the global re-balancing of aneuploid genomes Genome Biol 2010 5 216 10.1186/gb-2010-11-8-216 20804581 PMC2945780

[B37] Birchler JA Pal-Bhadra M Bhadra U Dosage dependent gene regulation and the compensation of the X chromosome in *Drosophila* males Genetica 2003 5 2–3 179 190 12723697 10.1023/a:1022935927763

[B38] Straub T Zabel A Gilfillan GD Feller C Becker PB Different chromatin interfaces of the *Drosophila* dosage compensation complex revealed by high-shear ChIP-seq Genome Res 2013 5 3 473 485 10.1101/gr.146407.112 23233545 PMC3589536

[B39] Erickson JW Quintero JJ Indirect effects of ploidy suggest X chromosome dose, not the X:A ratio, signals sex in *Drosophila* PLoS Biol 2007 5 12 e332 10.1371/journal.pbio.0050332 18162044 PMC2222971

[B40] Kacser H Burns JA The molecular basis of dominance Genetics 1981 5 3–4 639 666 7297851 10.1093/genetics/97.3-4.639 PMC1214416

[B41] Nordman J Orr-Weaver TL Regulation of DNA replication during development Development 2012 5 3 455 464 10.1242/dev.061838 22223677 PMC3252349

[B42] Duncan AW Hanlon Newell AE Smith L Wilson EM Olson SB Thayer MJ Strom SC Grompe M Frequent aneuploidy among normal human hepatocytes Gastroenterology 2012 5 New York: Elsevier 25 28 10.1053/j.gastro.2011.10.029 22057114 PMC3244538

[B43] Lee JT Bartolomei MS X-inactivation, imprinting, and long noncoding RNAs in health and disease Cell 2013 5 6 1308 1323 10.1016/j.cell.2013.02.016 23498939

[B44] Alekseyenko AA Ellison CE Gorchakov AA Zhou Q Kaiser VB Toda N Walton Z Peng S Park PJ Bachtrog D Kuroda MI Conservation and de novo acquisition of dosage compensation on newly evolved sex chromosomes in *Drosophila* Genes Dev 2013 5 853 858 10.1101/gad.215426.113 23630075 PMC3650223

[B45] Marin I Franke A Bashaw GJ Baker BS The dosage compensation system of *Drosophila* is co-opted by newly evolved X chromosomes Nature 1996 5 6596 160 163 10.1038/383160a0 8774878

[B46] Ellison CE Bachtrog D Dosage compensation via transposable element mediated rewiring of a regulatory network Science 2013 5 846 850 10.1126/science.1239552 24233721 PMC4086361

[B47] Arnold AP Itoh Y Melamed E A bird's-eye view of sex chromosome dosage compensation Annu Rev Genomics Hum Genet 2008 5 109 127 10.1146/annurev.genom.9.081307.164220 18489256

[B48] Hillier LW Miller W Birney E Warren W Hardison RC Ponting CP Bork P Burt DW Groenen MAM Delany ME Dodgson JB Chinwalla AT Cliften PF Clifton SW Delehaunty KD Fronick C Fulton RS Graves TA Kremitzki C Layman D Magrini V McPherson JD Miner TL Minx P Nash WE Nhan MN Nelson JO Oddy LG Pohl CS Randall-Maher J Sequence and comparative analysis of the chicken genome provide unique perspectives on vertebrate evolution Nature 2004 5 695 716 10.1038/nature03154 15592404

[B49] Adams MD Celniker SE Holt RA Evans CA Gocayne JD Amanatides PG Scherer SE Li PW Hoskins RA Galle RF George RA Lewis SE Richards S Ashburner M Henderson SN Sutton GG Wortman JR Yandell MD Zhang Q Chen LX Brandon RC Rogers YH Blazej RG Champe M Pfeiffer BD Wan KH Doyle C Baxter EG Helt G Nelson CR The genome sequence of *Drosophila melanogaster* Science 2000 5 5461 2185 2195 10.1126/science.287.5461.2185 10731132

[B50] Stein LD Bao Z Blasiar D Blumenthal T Brent MR Chen N Chinwalla A Clarke L Clee C Coghlan A Coulson A D'Eustachio P Fitch DHA Fulton LA Fulton RE Griffiths-Jones S Harris TW Hillier LW Kamath R Kuwabara PE Mardis ER Marra MA Miner TL Minx P Mullikin JC Plumb RW Rogers J Schein JE Sohrmann M Spieth J The genome sequence of *Caenorhabditis briggsae*: a platform for comparative genomics PLOS Biol 2003 doi:10.1371/journal.pbio.0000045 10.1371/journal.pbio.0000045 PMC261899 14624247

[B51] Venter JC Adams MD Myers EW Li PW Mural RJ Sutton GG Smith HO Yandell M Evans CA Holt RA Gocayne JD Amanatides P Ballew RM Huson DH Wortman JR Zhang Q Kodira CD Zheng XH Chen L Skupski M Subramanian G Thomas PD Zhang J Gabor Miklos GL Nelson C Broder S Clark AG Nadeau J McKusick VA Zinder N The sequence of the human genome Science 2001 5 5507 1304 1351 10.1126/science.1058040 11181995

[B52] Besson V Brault V Duchon A Togbe D Bizot JC Quesniaux VF Ryffel B Hérault Y Modeling the monosomy for the telomeric part of human chromosome 21 reveals haploinsufficient genes modulating the inflammatory and airway responses Hum Mol Genet 2007 5 17 2040 2052 10.1093/hmg/ddm152 17591625

[B53] Naurin S Hansson B Bensch S Hasselquist D Why does dosage compensation differ between XY and ZW taxa Trends Genet 2010 5 15 20 10.1016/j.tig.2009.11.006 19963300

[B54] Charlesworth D Charlesworth B Marais G Steps in the evolution of heteromorphic sex chromosomes Heredity (Edinb) 2005 5 118 128 10.1038/sj.hdy.6800697 15931241

[B55] Charlesworth B The evolution of chromosomal sex determination and dosage compensation Curr Biol 1996 5 149 162 10.1016/S0960-9822(02)00448-7 8673462

